# Availability of Adequate Photoprotection for Skin of Color

**DOI:** 10.7759/cureus.42794

**Published:** 2023-08-01

**Authors:** Yonesha Cole, Amber M Ilyas, Erum N Ilyas

**Affiliations:** 1 Dermatology, Drexel University College of Medicine, Philadelphia, USA; 2 Health and Environmental Impact, AmberNoon LLC, King of Prussia, USA

**Keywords:** tinted sunscreen, iron oxides, photoprotection, skin of color, sun protection

## Abstract

Introduction: The impact of ultraviolet (UV) light on the skin is well-established to have both immediate and delayed effects with increasing awareness of the impact of visible light (VL) on the skin with regard to photoaging and dyspigmentation. The effects of VL disproportionately impact the skin of color with regard to discoloration of the skin leading to guidelines for photoprotection that include the use of iron oxides in sunscreen products that impart a tint.

Methods: Commercially available sunscreen products found in the suncare and sun protection displays at local stores, pharmacies, and grocery stores were cataloged, and ingredients were reviewed for the presence of iron oxides.

Results: Of the 410 commercially available sunscreen products cataloged, 1.7% of facial sunscreen products available offered iron oxides, and 0.5% of non-lip products offered shade matching.

Conclusion: With only 1.7% of commercially available facial sunscreen products offering iron oxides in addition to traditional broad-spectrum UV protection, there is a clear gap in the market for iron oxide-containing sunscreen products to meet the photoprotection needs for the skin of color.

## Introduction

The impact of ultraviolet (UV) light on the skin is well-established to have both immediate and delayed effects. Tanning, sunburns, skin cancer, immunosuppression, actinic damage, and discoloration can be triggered without adequate UV protection [1]. In recent years, the impact of visible light (VL) on the skin with regard to photoaging and dyspigmentation has been noted with traditional sun-protective measures such as sunscreens that are not considered effective against these wavelengths of light [2,3].

VL is commonly encountered from sunlight exposure but is not the only source. Wavelengths for VL range from 400 to 700 nm with high-energy visible light (HEV), also known as blue light, in the 400-500 nm spectrum [4]. Light-emitting diode (LED) bulbs, fluorescent lighting, and electronic devices can also emit blue light. Although blue light has lower energy than UV light, it is capable of reaching the dermis up to 1 mm in depth [5,6]. Blue light has been shown to have a biological effect on the skin including indirect damage to DNA through oxidative stress [7]. This can trigger erythema and pigmentation in the skin of color [8].

The classification of skin types based on tanning potential in response to UV light in skin are referred to as Fitzpatrick skin types with skin types I through III demonstrating a tendency to burn easily with less of a potential to tan, while skin types IV through VI rarely burn with a higher tanning potential [9]. Reference to “skin of color” has been defined with regard to the skin’s response to UV light as Fitzpatrick skin types IV through VI based on the limited potential to burn easily in response to UV exposure. There are limitations to defining skin of color based on Fitzpatrick skin types; however, it is a well-known classification system to dermatologists and is the one referenced in studies with regard to the impact of blue light on the skin [10].

The skin of color may be disproportionately impacted by the effects of blue light. Campiche et al. demonstrated erythema and increased pigmentation induced by exposure to blue light in volunteers with Fitzpatrick skin types III and IV [4]. A study by Ramser et al. showed that in Fitzpatrick skin types IV through VI, blue light-induced hyperpigmentation was longer lasting [11]. Godley et al. tested the impact of blue light on epithelial cells without specifying the skin type and found that the development of reactive oxygen species upon exposure to blue light can lead to DNA damage and subsequent cellular dysfunction and potentially lead to the development of photoaging and tumorigenesis [12]. These findings highlight the need for protection not only against UV light but for other aspects of the electromagnetic spectrum.

Guidelines for photoprotection have evolved over the past decade as the impact of UV light and VL on the skin has been increasingly noted. In 2021, an expert panel provided guidelines for photoprotection based on skin types, recognizing the role of VL for skin of color in particular [13]. In 2022, consensus guidelines entitled “Photoprotection for skin of all color: consensus and clinical guidance from an expert panel” were released by a panel of dermatologists in the *Journal of the American Academy of Dermatology* with more specific recommendations [14]. Both sources noted the particular need for photoprotection in the skin of color that addresses VL in addition to traditional ultraviolet A (UVA) and ultraviolet B (UVB) protection as well as the possible role of anti-inflammatory agents, specifically licochalcone and glycyrrhetinate. More specifically, there is a need for photoprotection that considers shade matching to increase compliance in darker skin tones, which are disproportionately negatively impacted by VL and UVA more than lighter skin tones [13,14].

In light of recommendations for adequate photoprotection for skin of color that considers both broad-spectrum UV coverage along with VL protection, the question arose regarding the availability of options for consumers to purchase to satisfy the guidelines offered by clinicians easily. This study sought to determine (1) the availability of sunscreens that offer iron oxides for VL coverage in addition to traditional UVB protection distributed by major retail, grocery, and pharmacies in the United States (Target, Walgreens, CVS, and Wegmans) and (2) the presence of the anti-inflammatory agents such as licochalcone and glycyrrhetinate in these formulations.

## Materials and methods

It was determined that the study did not involve the use of human subjects, and IRB approval was not necessary. The suncare and sun protection displays at the local physical locations of four major retail pharmacies, grocery, and discount department stores (CVS, Walgreens, Target, and Wegmans) were visited during the dates of June 1 through June 10, 2023, in the Philadelphia region. The location of sunscreen products in each of the stores was identified under major displays for sun protection and suncare in addition to website reviews of each sunscreen manufacturer found in each store to completely catalog the full line of sunscreen products available to consumers in these outlets.

The available sunscreens, both in-store and online, for the brands found were cataloged by (1) name brand, (2) type (lotion, spray, stick, or lip balm), (3) sun protection factor (SPF), (4) claims for broad-spectrum, (5) active sunscreen ingredients, (6) inactive ingredients, (7) the presence or absence of iron oxides, (8) product claim for tint, (9) availability of varying hues if tint present, and (10) the presence or absence of licochalcone and/or glycyrrhetinate in the ingredient list. After cataloging the available products at local stores, websites for each brand were reviewed to ensure that each product available in each brand was included, given the variability that may exist regionally for in-store inventory.

Makeup-related cosmetic products used for the purpose of pigmentation such as concealers, foundations, and powders were excluded, given that the intent for use of these products tends to be traditional cosmetic use and not photoprotection.

For tinted sunscreens that are not considered makeup or cosmetic with multiple hues, only one hue was included in tallying the total number of available products; however, the number of hues available for each tinted sunscreen variety was cataloged.

After the sunscreen products were cataloged, they were screened for specific ingredients and criteria designated as photoprotection recommendations for skin of color. Products were filtered for (1) broad-spectrum, (2) presence of iron oxides, (3) claim for tint, (4) availability of varying hues, and (5) presence of licochalcone and/or glycyrrhetinate. We reviewed the active and inactive ingredients of all formulations for the presence of ingredients that offer UVB, UVA, and VL protection as well as anti-inflammatory agents, licochalcone and glycyrrhetinate. Basic descriptive statistics were performed.

## Results

A total of 410 sunscreen varieties were cataloged and reviewed. The majority of listed sunscreens, 380 out of 410 sunscreens cataloged (92.7%), offered an SPF of 30 or higher. All (100%) of the sunscreens evaluated offered broad-spectrum coverage. Only eight out of 410 sunscreen products (2.0%) offered a tint, and these same eight out of 410 sunscreens (2.0%) were the only sunscreens with iron oxides listed on the ingredient label (Table 1). No generic products were found with tint or iron oxides offered.

**Table 1:** Sunscreen products evaluated based on claims, SPF > 30, generic, presence of a tint as a product claim, the presence of iron oxides on the ingredient label, and the presence of both tint and iron oxides on the ingredient label SPF: Sun protection factor.

Sunscreen products evaluated	410
Sunscreen products with claims of broad-spectrum	410
Sunscreen products with an SPF > 30	380
Generic sunscreen products	76
Generic sunscreen products with SPF > 30	75
Sunscreen products with tint offered	8
Sunscreen products with iron oxides	8
Sunscreen products with a tint that included iron oxides in ingredients	8
Generic sunscreen products with a tint	0
Generic sunscreen products with iron oxides	0

Further evaluation of the eight sunscreen products available with iron oxides listed under ingredients revealed that of these eight products, all (100%) offered a tint as a product claim. Seven of the eight sunscreen products (87.5%) with a tint provided an SPF > 30. The product with an SPF < 30 was a lip balm compared to the other seven products listed as facial products in the form of lotions and a stick. Of the seven remaining tinted products, all offered an SPF > 30, and all seven (100%) were specified for face use. Only two of the seven (28.6%) non-lip products offered varying hues for shade matching, and both were specified for use on the face. All of the tinted sunscreen products (100%) with an SPF > 30 contained only inorganic sunscreen ingredients including zinc oxide and/or titanium dioxide. None (0%) of the tinted sunscreen products offered the antioxidant ingredients, licochalcone or glycyrrhetinate (Tables 2, 3).

**Table 2:** Tinted sunscreen products evaluated based on SPF, iron oxides on the ingredient label, use of organic or inorganic filters, and presence of antioxidants SPF: Sun protection factor.

Products with iron oxides	8
Products with iron oxides that stated “tint”	8
Products with iron oxides with SPF > 30	7
Products with iron oxides with varying hues offered	2
Products with iron oxides and inorganic UV filters	7
Products with iron oxides and organic UV filters	1
Products with iron oxides and titanium dioxide > 5%	4
Products with iron oxides and zinc oxide > 5%	3
Generic products with iron oxides	0
Products with iron oxides + licochalcone	0
Products with iron oxides + glycyrrhetinate	0

**Table 3:** Tinted sunscreen products available by brand, water resistance, varying hues, type, SPF, active ingredients, and ingredients SPF: Sun protection factor; PEG: Polyethylene glycol; EDTA: Ethylenediaminetetraacetic acid.

Brand	Water resistance	Varying hues	Type	SPF	Ingredients	Other ingredients
Sun Bum SPF 15 Sunscreen Tinted Lip Balm	Not stated	Not stated	Lip balm	15	Avobenzone 1.9%, Octinoxate 7.1%, Octisalate 4.9%	*Cocos nucifera* (coconut) oil, beeswax, *Butyrospermum parkii *(shea) butter, *Ricinus communis *(Castor) seed oil, *Olea europaea *(olive) fruit oil, caprylic/capric triglyceride, *Theobroma grandiflorum *seed butter, *Copernicia cerifera *(Carnauba) wax, flavor, *Helianthus annuus *(sunflower) seed oil, titanium dioxide, carmine, *Limnanthes alba *(Meadowfoam) seed oil, *Aloe barbadensis *leaf extract, *Simmondsia chinensis *(Jojoba) seed oil, *Carthamus tinctorius *(Safflower) seed oil, *Euphorbia cerifera *(Candelilla) wax, tocopherol, Jojoba esters, Octyldodecyl oleate, *Stevia rebaudiana *extract, iron oxides, mica, silica [15]
CeraVe Hydrating Mineral Sunscreen Face Sheer Tint SPF 30	No	No	Lotion	30	Titanium dioxide 5.5%, Zinc oxide 10%	Water, C12-15 alkyl benzoate, isohexadecane, isononyl isononanoate, dicapryl ether, PEG-30 dipolyhydroxystearate, triethylhexanoin, polyglyceryl-4 isostearate, dicaprylyl carbonate, ethylene/acrylic acid copolymer, triethanolamine, silica, poly C10-30 alkyl acrylate, stearic acid, ceramide NP, ceramide AP, ceramide EOP, carbomer, niacinamide, Cetearyl alcohol, triethoxycaprylsilane, behentrimonium methosulfate, sodium chloride, salicylic acid, sodium hyaluronate, sodium laurolyl lactylate, cholesterol, aluminum stearate, alumina, aluminum hydroxide, iron oxides, phenoxyethanol, P-anisic acid, chlorphenesin, tocopherol, disodium EDTA, disodium stearoyl glutamate, propylene carbonate, citric acid, caprylyl glycol, capryloyl salicylic acid, caprylic/capric triglyceride, diethylhexyl syringylidenemalonate, disteardimonium hectorite, xanthan gum, phytosphingosine, polyhydroxystearic acid, ethylhexylglyderin [16]
La Roche Posay *Anthelios *Mineral Tinted Sunscreen for Face SPF 50	40 minutes	No	Lotion	50	Titanium dioxide 11%	Water, isododecane, C12-15 alkyl benzoate, dimethicone, undecane, triethylhexanoin, isohexadecane, styrene/acrylates copolymer, nylon-12, caprylyl methicone, butyloctyl salicylate, phenethyl benzoate, silica, tridecane, dicaprylyl carbonate, dicaprylyl ether, talc, dimethicone/PEG 10/15 crosspolymer, aluminum stearate, pentylene glycol, PEG-9 polydimethylsiloxyethyl dimethicone, iron oxides, alumina, polyhydroxystearic acid, phenoxyethanol, magnesium sulfate, propylene glycol, caprylyl glycol, aluminum hydroxide, PEG-8 laurate, stearic acid, disteardimonium hectorite, diethylhexyl syringylidenemalonate, tocopherol, propylene carbonate, *Cassia alata *leaf extract, maltodextrin, benzoic acid, disodium stearoyl glutamate [17]
Neutrogena Mineral UV Tint for Face	80 minutes	Yes	Lotion	30	Titanium dioxide 3.2%, Zinc oxide 21.6%	Water, isohexadecane, dicaprylyl carbonate, dimethicone, isopropyl palmitate, isononyl isononanoate, cetyl PEG/PPG-10/1 dimethicone, C12-15 alkyl benzoate, sodium chloride, polyhydroxystearic acid, tocopheryl acetate, triethoxycaprylylsilane, sorbitan sesquioleate, phenoxyethanol, ethylhexylglycerin, dimethiconol, aluminum hydroxide, dimethicone crosspolymer, stearic acid, xanthan gum, iron oxides [18]
Everyday by Unsun Mineral Tinted Face Sunscreen Lotion	30 minutes	No	Lotion	30	Titanium dioxide 3%, Zinc oxide 3%	Water (aqua), isoamyl laurate, glycerin, polyglyceryl-2 ricinoleate, coconut alkanes, polyglyceryl-3 ricinoleate, coco-caprylate/caprate, *Crambe abyssinica* seed oil, sorbitan sesquioleate, heptyl undecylenate, *Aloe barbadensis *leaf juice, *Butyrospermum parkii *(Shea) butter, *Cocos nucifera *(coconut) oil, *Carthamus tinctorius *(safflower) seed oil, beeswax, *Olea europaea *(olive) fruit oil, tocopheryl acetate, hydrolyzed corn starch, *Beta vulgaris *(beet) root extract, *Citrus reticulata *(tangerine) fruit extract, *Citrus aurantium *(bitter orange) fruit extract, *Citrus sinensis* (sweet orange) peel extract, *Cucumis sativus *(cucumber) fruit extract, citric acid, lactic acid, tocopherol, magnesium sulfate, sodium chloride, triethoxycaprylylsilane, sodium glycerophosphate, benzyl alcohol, iron oxides (CI 77491, CI 77492, & CI 77499) [19]
Sun Bum Mineral Tinted 30 Face Lotion	40 minutes	No	Lotion	30	Titanium dioxide 5.8%, Zinc oxide 2.4%	Cyclopentasiloxane, isopropyl myristate, polyamide-5, dimethicone crosspolymer, stearalkonium hectorite, polysilicone-11, *Oryza sativa *(rice) bran extract, *Rosmarinus officinalis *(rosemary) leaf extract, *Helianthus annuus *(sunflower) extract, tocopherol, mica, iron oxides, propylene carbonate, synthetic wax, hydrogen dimethicone, isopropyl titanium triisostearate, aluminum hydroxide, silica silylate, octyldodecyl oleate [20]
Sun Bum Signature 30 Sunscreen Face Stick Tinted	80 minutes	No	Stick	30	Titanium dioxide 20.8%	Butyloctyl salicylate, hydrogenated polydecane, methyl methacrylate crosspolymer, dimethicone, caprylyl methicone, polyethylene, aluminum hydroxide, kaolin, microcrystalline wax, phytosteryl/behenyl/octyldodecyl lauroyl glutamate, isostearic acid, copernicia cerifera (carnauba) wax, C12-15 alkyl benzoate, tocopheryl acetate, iron oxides [21]
Australian Gold Botanical SPF 50 Tinted Face Sunscreen Lotion	80 minutes	Yes	Lotion	50	Titanium oxide 4%, Zinc oxide 4%	Cyclopentasiloxane, water/aqua/EAU, glycerin, silica, Cetyl PEG/PPG-10/1 dimethicone, disteardimonium hectorite, polymethylsilsesquioxane, PEG-10 dimethicone, hexyl laurate, polyglyceryl-4 isostearate, *Terminalia ferdinandiana *(Kakadu plum) fruit extract, *Eucalyptus globulus *(Eucalyptus) leaf extract, *Porphyra umbilicalis *extract, dimethicone crosspolymer, stearic acid, caprylyl glycol, tocopheryl acetate, *Butyrospermum parkii *(Shea) butter, panthenol, squalane, disodium EDTA, triethoxycaprylylsilane, alumina, phenoxyethanol, iron oxides (CI 77492, CI 77491, CI 77499) [22]

Out of the 410 varieties of sunscreen reviewed, seven tinted sunscreens were found for use on the face (1.7%) with none of these specified for use on the body as well (0%). Two of the 410 (0.5%) sunscreen products reviewed offered shade matching, and both were specifically for facial use. All seven of the tinted sunscreens available for face use used inorganic sunscreen ingredients with none using organic sunscreen ingredients.

## Discussion

Our results suggest that the availability of tinted sunscreen products to address the photoprotection needs of skin of color is lacking. With 410 varieties of sunscreen commercially available, only 1.7% were found to meet the guidelines recommended for photoprotection in skin of color for facial use with only 0.5% including product offerings for shade matching for varying hues of skin tone. There were no products designated for body use.

According to a research letter published in the *Journal of the American Academy of Dermatology* in 2021 entitled “A practical guide to tinted sunscreen,” Torres et al. recommended clinicians advise patients to “consider one’s skin tone and undertone when choosing the correct shade of tinted sunscreen” and that “sunscreen should be applied as the last step in skincare, before makeup (if desired)” [23]. However, from our review of 410 sunscreens available, only two products offer the option for shade matching. This makes it difficult for patients to translate clinician advice into practice as they have little choice in the selection process without being asked to consider the use of traditional makeup or cosmetic products to fill their needs. Practically speaking, even dermatologists have varying views on the use of traditional makeup products for the purpose of camouflaging during acne management, for example. A study of Italian dermatologists found that only half considered the routine use of cosmetic products acceptable [24]. Social barriers to the use of traditional cosmetic products exist in a study evaluating patients' perceptions of acne, which showed that female patients were more likely than male patients to use camouflage for acne scarring. This study also showed that 46% of those surveyed believed makeup to be an aggravating factor for acne [25]. Given these possible barriers to the use of makeup products for cosmetic usage, sunscreen products with an added tint may have the potential to increase compliance for use among a broader demographic of patients seeking photoprotection without the use of traditional cosmetics.

When recommending photoprotection, it is important to note that not all ingredients are created equal. A study by Beasley et al. compared the UVA protection offered by products containing titanium dioxide versus zinc oxide or avobenzone [26]. This study demonstrated that titanium dioxide did not offer sufficient protection from UVA in addition to noting the benefit of avobenzone concentrations of at least 3% and zinc oxide concentrations of 5% for adequate UVA protection. Iron oxides included in photoprotection offer protection from aspects of the electromagnetic spectrum and reduce the damage to the skin from VL [27]. It is important to note that iron oxides are listed on ingredient labels under ingredients without a specified concentration. Of the sunscreen products evaluated, the tinted sunscreen product intended for lip use included organic sunscreen filters such as avobenzone. With a concentration of avobenzone of 1.9%, this product did not appear to offer adequate UVA protection. The facial sunscreen products offered inorganic sunscreen filters with only half offering zinc oxide concentrations of >5% suggesting the possibility of inadequate UVA protection.

Although photoprotection recommendations for skin of color suggested the potential benefit of licochalcone and glycyrrhetinate [8], none of the tinted sunscreen products evaluated contained these ingredients.

Interestingly, all of the facial tinted sunscreen products available were exclusively based on inorganic active ingredients such as zinc oxide and/or titanium dioxide. The absence of a tint can create a white cast on the skin making these less desirable to use for skin of color [28]. Adding a tint to sunscreen can potentially increase compliance for use. However, given the lack of tinted sunscreen products using organic filters such as avobenzone for facial use, this suggests that the tint added to sunscreen products available is to increase compliance for the use of mineral sunscreen products by addressing the white cast created by these ingredients and not necessarily for VL protection. Weig et al. found that the appearance of sunscreen on the skin was considered a barrier to use in 33.7% of patients surveyed [29]. By recognizing a wider range of photoprotection that tinted sunscreen products offer beyond adding the tint simply for the purpose of camouflaging the white case created by inorganic filters, the use of organic sunscreen filters in tinted sunscreen products could offer consumers a wider range of options to choose from.

Due to the wide range of hues seen in the skin of color, with a limited number of products commercially available at large retail pharmacies, grocery stores, and discount department stores, there is minimal access to the available tinted sunscreen products to offer adequate photoprotection and matching hues. Although there are numerous products on the market that offer photoprotection, the majority of the current offerings fail to meet the recommendations of skin health experts for skin of color.

Limitations of our study include the evaluation of only four major US retailers, which may not be fully representative of the retailers frequented in different regions of the United States. However, website reviews of each brand represented in these retailers were performed to add new products discovered while reviewing each manufacturer's website to include variations of each product available. Another limitation of our study is not including larger beauty outlets and department stores in our search. However, given that availability and cost are key considerations for clinicians to offer practical advice to patients for product recommendations, focusing on products available at an accessible price point was important to recognize the challenges that patients face in following care instructions. Weig et al. found that 16.4% of patients surveyed identified cost as a barrier to the use of sunscreen products [29]. This underscores how essential affordable photoprotection options are for clinicians to provide recommendations with which patients can comply. Lastly, although there are likely a variety of traditional makeup products such as concealers and foundation that may offer iron oxides in addition to UV protection, these products were specifically excluded, given that the primary intent for use tends to be for cosmetic usage as opposed to routine photoprotection. The intent for use has the potential to create a barrier to widespread use based on the perception of these products for cosmetic purposes rather than true photoprotection.

## Conclusions

There is a clear gap in the market for tinted sunscreen products to meet the photoprotection needs for skin of color. Tinted sunscreen products have the potential to offer broad-spectrum coverage for UV light and VL with a wide range of benefits, in particular for skin of color. Products that offer shade matching have the potential to increase compliance. Although guidelines for photoprotection in the skin of color clearly indicate the need for tinted sunscreen options, there is a need for manufacturers to provide options to match clinician recommendations.
